# Application of 5-aminolevulinic acid promotes ripening and accumulation of primary and secondary metabolites in postharvest tomato fruit

**DOI:** 10.3389/fnut.2022.1036843

**Published:** 2022-11-10

**Authors:** Junwen Wang, Hong Yuan, Yue Wu, Jihua Yu, Basharat Ali, Jing Zhang, Zhongqi Tang, Jianming Xie, Jian Lyu, Weibiao Liao

**Affiliations:** ^1^College of Horticulture, Gansu Agricultural University, Lanzhou, China; ^2^State Key Laboratory of Aridland Crop Science, Gansu Agricultural University, Lanzhou, China; ^3^Department of Agricultural Engineering, Khwaja Fareed University of Engineering and Information Technology, Rahim Yar Khan, Pakistan

**Keywords:** 5-aminolevulinic acid, postharvest tomato fruit, carotenoids metabolism, phenolic acid, flavonoid

## Abstract

5-Aminolevulinic acid (ALA) plays a vital role in promoting plant growth, enhancing stress resistance, and improving fruit yield and quality. In the present study, tomato fruits were harvested at mature green stage and sprayed with 200 mg L^–1^ ALA on fruit surface. During ripening, the estimation of primary and secondary metabolites, carotenoids, and chlorophyll contents, and the expression levels of key genes involved in their metabolism were carried out. The results showed that ALA significantly promoted carotenoids accumulation by upregulating the gene expression levels of *geranylgeranyl diphosphate synthase* (*GGPPS*, encoding geranylgeranyl diphosphate synthase), *phytoene synthase 1* (*PSY1*, encoding phytoene synthase), *phytoene desaturase* (*PDS*, encoding phytoene desaturase), and *lycopene*β*-cyclase* (*LCYB*, encoding lycopene β-cyclase), whereas chlorophyll content decreased by downregulating the expression levels of *Mg-chelatase* (*CHLH*, encoding Mg-chelatase) and *protochlorophyllide oxidoreductase* (*POR*, encoding protochlorophyllide oxidoreductase). Besides, the contents of soluble solids, vitamin C, soluble protein, free amino acids, total soluble sugar, organic acid, total phenol, and flavonoid were increased in ALA-treated tomato fruit, but the fruit firmness was decreased. These results indicated that the exogenous ALA could not only promote postharvest tomato fruit ripening but also improve the internal nutritional and flavor quality of tomato fruit.

## Introduction

As a major horticultural crop worldwide, tomato is highly praised by consumers all over the world due to its unique aroma and abundance of nutrients such as lycopene, organic acid, and vitamins (1, 2). Tomato fruit is also an important source of phenolic acids and flavonoids (3). Several studies have indicated that the intake of tomato fruit can delay aging and inhibit the occurrence of cancer (4, 5), so it has a crucial role in human health. Recently, consumers have had higher requirements for the internal nutrition, flavor, and appearance of tomato fruit, thus, improvement of the qualities of tomato fruit has become an important research area.

Fruit ripening is a highly complex process accompanied by various physiological and biochemical reactions. The formation process of tomato fruit quality is often accompanied by fruit ripening (6). In this process, carotenoid-dominated pigments are gradually accumulated, aroma volatiles are synthesized, and the conversion of complex carbohydrates into sugars (7). The accumulation of carotenoids (especially lycopene) is one of the most representative characteristics, which directly lead to the change of tomato fruit from green to red (8). In recent years, a considerable number of reports have focused on strategies to promote tomato fruit ripening and improve quality. For example, the use of plant growth regulators or signaling molecules to promote fruit ripening and improve quality is a convenient and cheap method (9). Plant hormones and regulators (such as abscisic acid and brassinosteroids) have been widely used in practical production to regulate fruit color and improve quality, thereby obtaining high-quality fruit (10, 11).

Among many identified plant growth regulators and phytohormones, 5-aminolevulinic acid (ALA) widely exists in animals and plants (12). It is an essential biosynthetic precursor of all tetrapyrrole compounds, including chlorophyll, heme, and phytochrome in higher plants (13). Previous studies have shown that ALA has the physiological activity of phytohormones (14). High concentration of ALA can be used as pollution-free and residue-free pesticides and herbicides (15). While a relatively low concentration of ALA could promote plant growth and enhance stress resistance, such as seed germination (16), root growth (17), tolerance to salt, and drought and heavy metal stress (18–20). In addition, ALA could regulate the internal nutritional qualities and appearance quality of fruit. For example, exogenous ALA application significantly increased vitamin C, soluble solids, and soluble sugar contents thus improving the nutritional quality of apple fruit (21). The content of anthocyanin in peach skin was increased after the application of ALA, resulting in improved peach coloration (12). ALA treatment significantly upregulated the expression of genes (*MdDFR*, *MdCHS*, *MdUFGT*, and *MdLDOX*) involved in anthocyanin synthesis in apple skin (9). These results suggested that ALA has the potential to regulate fruit ripening and promote coloring. In our recent study, the exogenous ALA significantly increases carotenoid biosynthesis by regulating the expression level of genes (*PSY1*, *PDS*, and *LCY-B*) and promotes early on-tree maturation of tomato fruit (22). These results confirm that ALA could not only promote color formation but also improve the internal nutritional qualities of fruit. However, in practical tomato production, mature green fruit is usually harvested for storage and transportation. In this harvest-to-sale link, the tomato fruit is often not fully ripe, and the fruit quality is not optimal when it is marketed for sale. Therefore, applying ALA in postharvest tomato fruit is a feasible strategy to promote fruit ripening and improve quality. In addition, there are few reports about the effects of ALA on carotenoids, sugar and acid compounds, and other secondary metabolites during postharvest ripening of tomato fruit. Therefore, in this study, the regulatory role of ALA on pigments, qualities, and other primary and secondary metabolites of postharvest tomato fruit was investigated. To further understand the mechanisms of ALA-induced fruit color change, the key gene expression levels involved in carotenoids and chlorophyll biosynthesis/metabolism were also assessed. Our results provide theoretical support for the application of ALA on postharvest tomato fruit, which regulates their coloring and quality.

## Materials and methods

### Plant materials and treatment

Tomato fruits (*Solanum lycopersicum* L. cv. Yuanwei No.1) were harvested at the mature green stage from a solar greenhouse in Yuzhong County, Lanzhou City, China (35.85° N, 104.09° E). A total of 150 fruits of uniform size, no visual blemishes, and consistent maturity were selected. These fruits were randomly divided into two groups, of which one for control and another for ALA treatment. All fruit were washed with deionized water and drained before treat.

### Aminolevulinic acid treatment group

tomato fruit surface was sprayed evenly with 200 mg L^–1^ ALA (Yuanye, China) solution, which contained 0.01% Tween-20 (Solarbio, China); the concentration of ALA used in this study is an appropriate concentration selected from our previous study (22). Control group: fruit surface was sprayed with an equal amount of deionic water to the ALA treatment group (also contained 0.01% Tween-20). The fruits were treated at 0, 4, and 8 days after harvest. During the treatment period, all fruits were placed in an artificial climate chamber (Dongnan Instrument Co., Ltd., China). The photoperiod condition was 12 h/12 h. When illuminating, the temperature was 25 ± 1^°^C, light intensity was 300 μmol s^–1^ m^–2^, and humidity was 70%. In the dark, the ambient temperature was 18 ± 1^°^C, and the relative humidity was 50%. Fruit samples were collected on the 0, 4, 8, and 12 days after the first treatment. The standard for fruit ripening stage was considered according to the description of Shinozaki et al. (23), that is, mature green stage (full-size green fruit), breaker stage (less than 10% green to orange), pink stage (100% pink), red ripe stage (full red), and over-ripening stage (full red, fruit softens seriously).

### Color parameters of tomato fruit skin

After being treated by ALA, the fruit was photographed each time after sampling. The color parameter (a*, the color change from green to red) of the tomato fruit was determined using a CR-10 Plus colorimeter (Konina Minolta, Japan). The color parameter was measured at three points (shoulder, equator, and top) of tomato fruit. Repeat three times for each treatment.

### Fruit quality attributes

The GY-4-J digital fruit firmness tester (Top Cloud-agri Technology, China) was used to determine the firmness of fresh tomato fruit. The content of soluble solids was determined by a refractometer (PAL-1, ATAGO Co., Ltd., Japan). The 2,6-dichloroindophenol stain method was used to determine vitamin C content (24). The coomassie brilliant blue G-250 staining method was used to determine the content of soluble protein (25). The free amino acid content was measured using ninhydrin assay method (22).

### Sugar and organic acid components

The extraction and determination of sugar component content were performed according to the previous method in our laboratory (26). A 5 g of fresh tomato fruit samples were homogenized. Ultrasonic oscillation at 30^°^C for 60 min, then centrifuged the samples at 4^°^C for 10 min at 8,500 × *g* 2 ml supernatant was added through a 0.22-μm aqueous filter. The determination of sugar component content used high-performance liquid chromatography (HPLC) with a refractive index detector (Agilent 1100, Agilent Technologies, United States). The chromatographic column: LC-NH2 amino column (250 × 4.6 mm, Phenomenex, United States); the mobile phase: acetonitrile and water (75: 25, v/v); flow rate: 1.0 ml min^–1^; column temperature: 30^°^C; and injection volume: 20 μl. The retention time of fructose, glucose, and sucrose (standards from Yuanye Bio-Technology Co., Ltd., China) was 6.6, 7.7, and 10.6 min, respectively. The sugar components were identified according to the retention time of standards.

The extraction and determination of organic acid component content were performed according to the previous methods in our laboratory (26). Fresh fruit samples (5 g) were homogenized, transferred into 25 ml centrifugal tube and make the volume constant, and then centrifuged at 8,500 × *g* for 10 min at 4^°^C. A 2-ml supernatant was added through a 0.22-μm aqueous filter, and the filtrate was used for HPLC with a UV detector (EMpower3 2998 AcQuITY Arc, Waters, United States), The chromatographic column: Hi-PiexH (300 × 7.7 mm, Agilent Technologies, United States); the detection wavelength: 210 nm; mobile phase: dihydrogen phosphate sodium (0.2 mmol L^–1^); flow rate: 0.5 ml min^–1^; column temperature: 25^°^C; and injection volume: 10 μl. The retention time of tartaric acid, quinic acid, malic acid, shikimic acid, citric acid, and succinic acid (standards from Yuanye Bio-Technology Co., Ltd., China) was 7.1, 7.6, 9.7, 11.0, 17.8, and 24.0 min, respectively. The organic acid components were identified according to the retention time of standards.

### Phenol and flavonoid compounds

The determination of total phenol content was performed according to the method of Hand et al. (27), with slight modifications. Fresh fruit sample (1 g) was weighed and transferred to a mortar with 0.2 ml 50% methanol and 4.8 ml deionized water, ground to homogenate, followed by a 3-ml mixture of 20% sodium carbonate and Folin-Ciocalteu’s phenol reagent (5:1, v/v). Extract under dark conditions for 30 min at 50^°^C. The absorbance of the extract was measured at 760 nm using a UV-1800 spectrophotometer (Shimadzu, Japan). The determination of flavonoid content was performed according to the method of Lin et al. (28), with slight modifications. Briefly, fresh fruit sample (1 g) was weighed and transferred to a test tube with 1 ml of deionized water. Take 0.5 ml of the solution and add 0.1 ml potassium acetate (1 mol L^–1^), 1.5 ml alcohol (95%), 0.1 ml aluminum chloride hexahydrate (10%), and 2.8 ml deionized water. Then extracted at 25^°^C for 40 min. The absorbance of the reaction mixture was measured at 415 nm using a UV-1800 spectrophotometer (Shimadzu, Japan).

For determination of the 11 phenols (protocatechuic acid, p-hydroxybenzoic acid, chlorogenic acid, gallic acid, 4-coumaric acid, ferulic acid, cinnamic acid, gentianic acid, caffeic acid, cynarin, and benzoic acid) and 4 flavonoids (rutin, quercetin, naringenin, and kaempferol) were performed according to the method of Wang et al. (26), with slight modifications. Take 0.1 g of freeze-dried tomato sample powder, transfer to a test tube (containing 2 ml methanol), and incubate at 4^°^C for 1 h. Then centrifuged at 5,500 × *g* at 4^°^C for 10 min, the supernatant was added through a 0.22-μm organic filter membrane, and finally analyzed by HPLC (Alliance Waters e2695, Waters, United States). HPLC column: C_18_ column (250 mm × 4.6 mm, 5 μm, Waters, United States); flow rate: 1.1 ml min^–1^; injection volume: 10 μl; column temperature: 30^°^C; and mobile phase: methanol and 1% acetic acid and gradient elution (Supplementary Table 1). The detection wavelength, retention time, and standards manufacturer of the identified compounds are shown in Supplementary Table 2. Data were analyzed with Empower Software (Waters, United States).

### Carotenoid compounds

Carotenoids were determined according to the method in our previous study (22). A 0.5 g freeze-dried tomato fruit sample powder was taken and 30 ml of a mixture of acetone and petroleum ether was added (1: 2, v/v). After ultrasonic extraction at 30^°^C for 40 min, the extract was dried at 40^°^C, then dissolved in a mixture of 6.25 ml methanol, 5 ml dichloromethane, and 13.75 ml acetonitrile. Subsequently, a 2-ml mixture was added through a 0.22-μm organic filter membrane, and the filtrate was used for an Alliance Waters e2695 HPLC (Waters, United States). The chromatographic column: C_18_ column (250 mm × 4.6 mm, 5 μm, Waters, United States), mobile phase was composed of methanol: dichloromethane: acetonitrile (25:20:50, v/v/v), flow rate: 1.2 ml min^–1^, injection volume: 10 μl, and column temperature: 30^°^C.

Phytoene (Sigma, United States) was detected at 286 nm, lutein and β-carotene (Yuanye, China) were detected at 450 nm, and lycopene (Yuanye, China) was detected at 470 nm. The retention time of lutein, phytoene, lycopene, and β-carotene was 2.7, 7.7, 8.3, and 12.7 min, respectively. Data were analyzed with Empower Software (Waters, United States).

### Intermediates contents on aminolevulinic acid metabolic pathway

The content of endogenous ALA was determined according to the previously reported method (20). Fresh fruit sample (3 g) was weighed and transferred to a mortar, added a 6-ml acetate buffer (pH = 4.6), ground to homogenate. After centrifuging at 5,000 × *g* for 15 min. After that, the supernatant (5 ml) was mixed with four drops of ethyl acetoacetate and incubated in boiling water. After cooling to room temperature, fresh Ehrlich’s reagent solution (containing 42 ml glacial acetic acid, 8 ml 70% perchloric acid, and 1 g dimethylaminobenzaldehyde) in the same volume was mixed and allowed for 15 min. The absorbance of the supernatant was determined at 554 nm.

The content of chlorophyll was determined according to the previously reported method (20). Fresh tomato fruit sample (2 g) was placed in a test tube with 10 ml 80% acetone, and then chlorophyll was extracted under avoided light conditions for 2 days. The absorbance of extract was determined at 646 and 663 nm.

The content of protochlorophyllide (Pchlide) was determined according to the previously reported method (20). Exactly, Fresh fruit (1 g) was homogenized and placed in a test tube, then 25 ml of alkaline acetone was added (80%). The homogenate was extracted under avoided light conditions for 2 days. After centrifuging at 1,500 × *g* for 10 min. The absorbance was measured using a UV-1800 spectrophotometer (Shimadzu, Japan).

### Total RNA extraction and relative gene expression levels

RNAprep Pure Plant Plus Kit and FastKing RT Kit (Tiangen Biotech Co., Ltd., China) were used to extract total RNA and synthesize cDNA, respectively, and performed following the methods described in the manufacturer’s instructions. A 1 μl of cDNA was used for quantitative real-time PCR (qRT-PCR) analysis in a real-time PCR detection system (QuantStudio 5 Real-Time PCR System; Thermo Fisher Scientific, United States). The internal control was *Actin* gene of tomato. The primers are listed in Supplementary Table 3. The relative expression levels of genes were determined using the comparative CT method (29).

### Statistical analyses

The data from all experiments were expressed as means ± SE. The Student’s *t*-test was used to determine significant differences between means using SPSS 23.0 (IBM Co., United States). The principal component analysis (PCA), correlation analysis, and figures were prepared with Origin 2022 (OriginLab Co., United States). Pearson’s correlation analysis was conducted to investigate correlations.

## Results

### Aminolevulinic acid promoted the quality of postharvest tomato fruit

During postharvest storage, the firmness of tomato fruit decreased gradually with the increase in maturity (Table 1). Compared with the control, ALA treatment stimulated these decreases. At 8 DAT, the fruit firmness of ALA treatment was significantly lower than that of the control. At 12 DAT, the fruit firmness of ALA treatment was significantly lower than that of the control. Soluble solids content showed an increasing trend with storage time. While, compared with the control, soluble solids content was higher in ALA-treated. Especially, at 8 DAT, the soluble solids in ALA-treated fruit were significantly higher than the control, but at 12 DAT, the soluble solids in ALA-treated fruit decreased. Vitamin C content in all fruits increased with storage time. Further, ALA-treated fruit also had higher vitamin C content compared with control fruit. The soluble protein content in control was increased from 0.37 to 0.97 g kg^–1^ during the storage. Compared with the control, ALA treatment significantly increased soluble protein content at 4 and 8 DAT, and at the end of storage, the soluble protein content in ALA-treated fruit was 1.03 g kg^–1^. In general, the trend of free amino acid content was the same as that of soluble protein content at 8 DAT, and free amino acid content attained the highest levels under ALA treatment at 8 DAT. However, the free amino acid content was significantly inhibited by ALA treatment at 12 DAT.

**TABLE 1:** Effects of ALA on quality attributes of postharvest tomato fruit.

Days after treatment (d)	Treatments	Firmness (kg cm^–^^2^)	Soluble solids (%)	Vitamin C (mg kg^–^^1^ FW)	Soluble protein (g kg^–^^1^ FW)	Free amino acid (g kg^–^^1^ FW)
0	Control	5.12 ± 0.49	4.03 ± 0.03	47.96 ± 1.99	0.37 ± 0.08	0.13 ± 0.0075
	ALA	5.54 ± 0.42	4.30 ± 0.17	43.73 ± 0.71	0.39 ± 0.06	0.15 ± 0.0044
4	Control	4.46 ± 0.54	5.40 ± 0.10	56.72 ± 3.01	0.62 ± 0.02	0.23 ± 0.0076
	ALA	5.21 ± 0.18	5.77 ± 0.07 *	57.98 ± 2.41	0.70 ± 0.01 *	0.21 ± 0.0076
8	Control	4.17 ± 0.14*	5.97 ± 0.12	80.25 ± 2.95	0.70 ± 0.02	0.22 ± 0.0029
	ALA	3.65 ± 0.12	6.63 ± 0.15*	97.37 ± 3.31*	0.84 ± 0.00*	0.24 ± 0.0058*
12	Control	2.76 ± 0.05*	6.07 ± 0.09*	89.58 ± 1.25	0.97 ± 0.03	0.30 ± 0.0107**
	ALA	2.42 ± 0.10	5.67 ± 0.09	97.63 ± 2.97	1.03 ± 0.04	0.22 ± 0.0058

The data represent mean ± SE from three independent replicates, and asterisks indicate a significant difference between the treatments using the Student’s *t*-test (*p < 0.05, **p < 0.01).

### Aminolevulinic acid regulated sugar and organic acid components of postharvest tomato fruit

**FIGURE 1:**
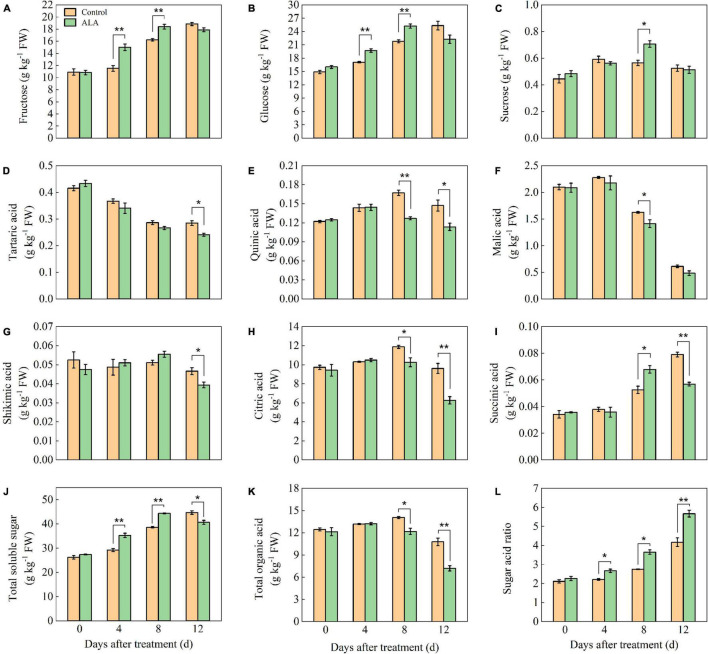
Effects of ALA on sugar and organic acid component content of postharvest tomato fruit. **(A)** The fructose content; **(B)** the glucose content; **(C)** the sucrose content; **(D)** the tartaric acid content; **(E)** the quinic acid content; **(F)** the malic acid content; **(G)** the shikimic acid content; **(H)** the citric acid content; **(I)** the succinic acid content; **(J)** the total soluble sugar content; **(K)** the total organic acid content; and **(L)** the sugar–acid ratio content. Vertical bars represent mean ± SE from three independent replicates, and asterisks indicate a significant difference between the treatments using the Student’s *t*-test (*p < 0.05, **p < 0.01).

At 4 and 8 DAT, the fructose content of ALA-treated fruit was significantly higher than that of the control (Figure 1A). At 8 DAT, the fructose content in ALA treatment reached a maximum level, which was 13.58% higher than that of the control. Concordant with fructose, ALA treatment significantly increased the content of glucose in the tomato fruit at 4 and 8 DAT (Figure 1B). After ALA treatment, the sucrose content in the fruit was 1.27-fold as high as that in the control group at 8 DAT (Figure 1C).

The tartaric acid content in tomato fruit gradually decreased with storage time (Figure 1D). The tartaric acid content in ALA treatment was significantly lower than that of the control at 12 DAT. The content of quinic acid in tomato fruit increased first and then decreased during storage (Figure 1E). At 8 and 12 DAT, the quinic acid content in ALA-treated fruit was significantly lower than that in the control. The malic acid content was decreased at the end of the storage in both ALA treated and untreated tomato fruit (Figure 1F). ALA treatment could maintain a lower level of malic acid as compared with control, especially at 8 DAT, the malic acid content in ALA-treated fruit was significantly decreased as compared to untreated fruit. ALA treatment had no effect on the content of shikimic acid in tomato fruit at the early of storage, but at 12 DAT, the ALA-treated fruit was significantly lower than that of the control (Figure 1G). At 8 and 12 DAT, the citric acid content of ALA-treated fruit decreased by 15.53 and 52.38% compared with the control, respectively (Figure 1H). From 0 to 8 DAT, the succinic acid content in ALA-treated fruit increased gradually (Figure 1I) and compared with the control, ALA treatment significantly improved the succinic acid content in tomato fruit at 8 DAT. However, ALA treatment significantly reduced the content of succinic acid in tomato fruit at 12 DAT.

After ALA treatment, the total sugar content of tomato fruit increased from 0 to 8 DAT and then decreased at 12 DAT (Figure 1J). In particular, the soluble total sugar content in ALA-treated fruit was significantly higher than that in the control at 4 and 8 DAT. At early storage time, there was no significant effect on total organic content among the treatments (Figure 1K). But the total organic acid content in fruit treated with ALA decreased at 8 and 12 DAT. Compared with the control, ALA application could significantly increase the sugar–acid ratio during storage (Figure 1L). At 4, 8, and 12 DAT, the sugar–acid ratio of ALA-treated fruit was 20.27, 32.73, and 35.97% higher than that of the control, respectively.

### Aminolevulinic acid regulated phenolic acid and flavonoid compounds of postharvest tomato fruit

**FIGURE 2:**
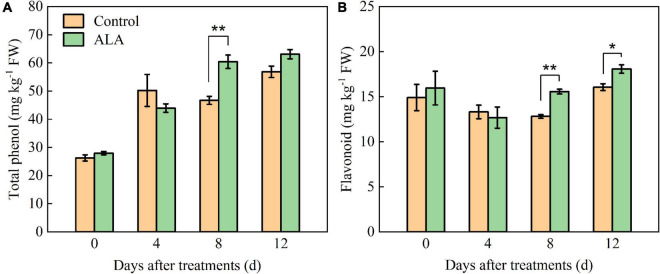
Effects of ALA on total phenol and flavonoid content of postharvest tomato fruit. **(A)** The total phenol content; and **(B)** the flavonoid content. Vertical bars represent mean ± SE from three independent replicates, and asterisks indicate a significant difference between the treatments using the Student’s *t*-test (*p < 0.05, **p < 0.01).

The ALA treatment had significant effects on the total phenol and flavonoid contents of tomato fruit (Figure 2). After ALA treatment, the total phenol content significantly increased by 29.36% compared with the control at 8 DAT (Figure 2A). Compared with the control at 8 and 12 DAT, the flavonoid content in tomato fruit was significantly increased by 21.45 and 12.52% after treatment with ALA (Figure 2B).

**FIGURE 3:**
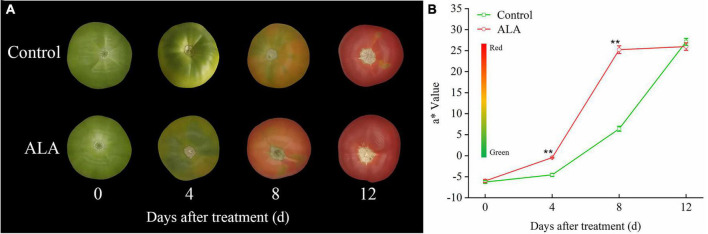
Effects of ALA on skin color parameters of postharvest tomato fruit. **(A)** The fruit color; **(B)** the a* value, the color bar next to the *Y*-axis gives an indication of the relation between a* value and fruit color, green to red indicates a* value from small to large. The data presented represent mean ± SE from three independent replicates, and asterisks indicate a significant difference between the treatments using the Student’s *t*-test (*p < 0.05, **p < 0.01).

**FIGURE 4:**
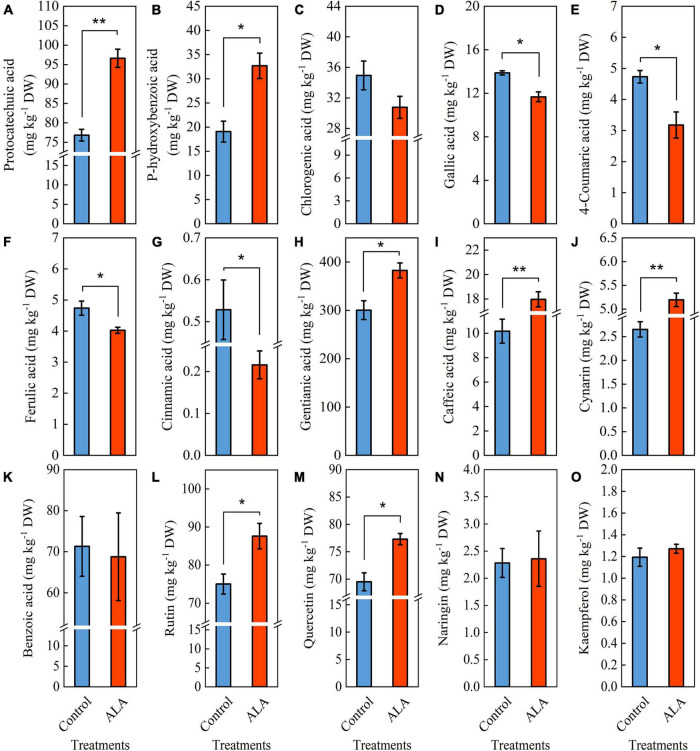
Effects of ALA on phenolic acid and flavonoid compounds content of postharvest tomato fruit. **(A–K)** Phenolic acid compounds and **(L–O)** flavonoid compounds. **(A)** The protocatechuic acid content; **(B)** the p-hydroxybenzoic acid content; **(C)** the chlorogenic acid content; **(D)** the gallic acid content; **(E)** the 4-coumaric acid content; **(F)** the ferulic acid content; **(G)** the cinnamic acid content; **(H)** the gentianic acid content; **(I)** the caffeic acid content; **(J)** the cynarin content; **(K)** the benzoic acid content; **(L)** the rutin content; **(M)** the quercetin content; **(N)** the naringin content; and **(O)** the kaempferol content. Vertical bars represent mean ± SE from three independent replicates, and asterisks indicate a significant difference between the treatments using the Student’s *t*-test (*p < 0.05, **p < 0.01).

The ALA treatment induced significant color changes at 8 DAT in tomato fruit (Figure 3A), and the contents of total phenol and flavonoid in tomato fruit treated with ALA increased significantly (Figure 2). Therefore, we further quantitatively analyzed 11 phenolic acid compounds (protocatechuic acid, p-hydroxybenzoic acid, chlorogenic acid, gallic acid, 4-coumaric acid, ferulic acid, cinnamic acid, gentianic acid, caffeic acid, cynarin, and benzoic acid) and 4 flavonoid compounds (rutin, quercetin, naringin, and kaempferol) by HPLC in tomato fruit at 8 DAT. Compared with the control, ALA treatment significantly increased the contents of five phenolic acids and two flavonoid compounds in postharvest tomato fruit, including protocatechuic acid (Figure 4A), p-hydroxybenzoic acid (Figure 4B), gentianic acid (Figure 4H), caffeic acid (Figure 4I), cynarin (Figure 4J), rutin (Figure 4L), and quercetin (Figure 4M). Contrastingly, the contents of gallic acid (Figure 4D), 4-coumaric acid (Figure 4E), ferulic acid (Figure 4F), and cinnamic acid (Figure 4G) in tomato fruit were decreased after ALA treatment. In addition, there was no difference between the two phenolic acid compounds [chlorogenic acid (Figure 4C) and benzoic acid (Figure 4K)] and two flavonoid compounds [naringin (Figure 4N) and kaempferol (Figure 4O)] in tomato fruit treated with ALA and the control group.

### Aminolevulinic acid accelerated coloring of postharvest tomato fruit

In this study, ALA treatments induced significant color changes. At 4 DAT, the fruit treated with ALA reached the breaker stage. At 8 DAT, the tomato fruit skin color with ALA treatment was light red, while the skin color of the control tomato fruit was still mostly green. At 12 DAT, all fruits had become fully red, but there was no difference in color (Figure 3A). However, the fruit firmness of ALA treatment was significantly lower than that of the control (Table 1), and the fruit has been seriously softened. Therefore, we believe that the fruit of ALA treatment has been over-ripening at 12 DAT. Accordingly, treatment with ALA significantly accelerated the transition from green to red compared to control. In particular, at 4 and 8 DAT, a* value of ALA-treated fruit was significantly higher than that of the control (Figure 3B).

### Aminolevulinic acid affected carotenoids and intermediates of chlorophyll biosynthesis of postharvest tomato fruit

**FIGURE 5:**
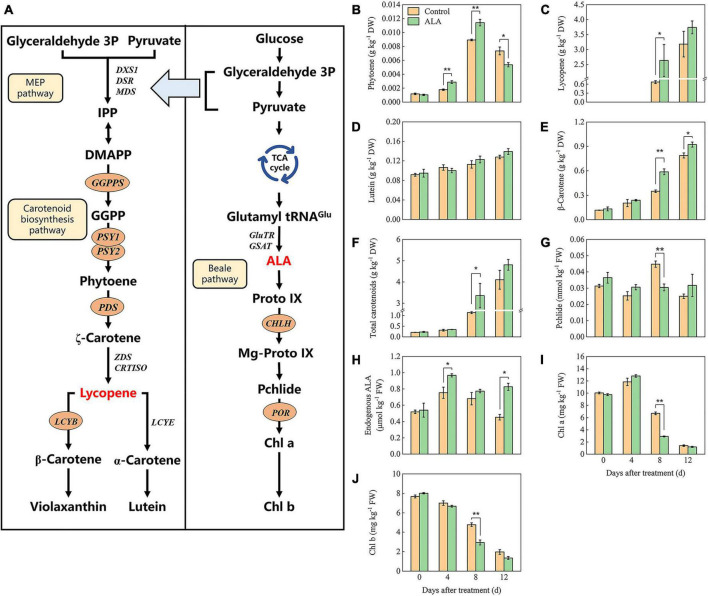
Effects of ALA on carotenoids and intermediates of chlorophyll biosynthesis content of postharvest tomato fruit. **(A)** The carotenoids and chlorophylls biosynthesis pathway (67, 68, 75); **(B)** the phytoene content; **(C)** the lycopene content; **(D)** the lutein content; **(E)** the β-carotene content; **(F)** the total carotenoids content; **(G)** the pchlide content; **(H)** the endogenous ALA content; **(I)** the Chl *a* content; and **(J)** the Chl *b* content. Vertical bars represent mean ± SE from three independent replicates, and asterisks indicate a significant difference between the treatments using the Student’s *t*-test (*p < 0.05, **p < 0.01).

Carotenoid synthesis and metabolism pathways have been identified (Figure 5A); to further investigate the influence of ALA treatment on the color of postharvest tomato fruit, carotenoids (phytoene, lycopene, lutein, β-carotene, and violaxanthin) contents were analyzed. The content of phytoene first increased and then decreased with storage (Figure 5B). Phytoene content in fruit treated with ALA was significantly higher than that of the control by 4 and 8 DAT but decreased on the 12 DAT. Lycopene was not detected in either ALA-treated or untreated fruit at the early of storage (0 and 4 DAT). A small amount of lycopene was detected in the fruit of the control group at 8 DAT, which was significantly lower than that of ALA treatment (Figure 5C). However, at the end of the storage period, there was no difference in all fruit. At early storage duration, carotenoids in all fruit mainly included lutein and β-carotene. The content of lutein and β-carotene increased gradually with storage (Figures 5D,E). β-Carotene content in the fruit of ALA treatment was significantly higher than that of the control at 8 and 12 DAT. Similarly, ALA treatment significantly increased the accumulation of total carotenoids in tomato fruit (Figure 5F).

The contents of several important intermediates among the ALA downstream metabolic pathway were determined to investigate the effect of ALA on the endogenous ALA and chlorophyll contents in tomato fruit. As shown in Figure 5H, at 4 DAT, endogenous ALA content in tomato fruit treated with ALA increased significantly. Subsequently, endogenous ALA content decreased with the passage of storage time. At 12 DAT, the endogenous ALA content in the ALA treatment group was significantly enhanced. Under ALA treatment, the Pchlide content of the fruit initially increased and then later decreased (Figure 5G). At 8 DAT, the content of pchlide in fruit under ALA treatment was significantly lower than those of the control group. Chlorophyll content decreased gradually with storage time (Figures 5I,J). On the 8 DAT, for ALA treatment, Chl *a* and Chl *b* contents were significantly decreased compared with the control.

### Aminolevulinic acid regulated gene expressions of carotenoids metabolism and chlorophyll biosynthesis of postharvest tomato fruit

**FIGURE 6:**
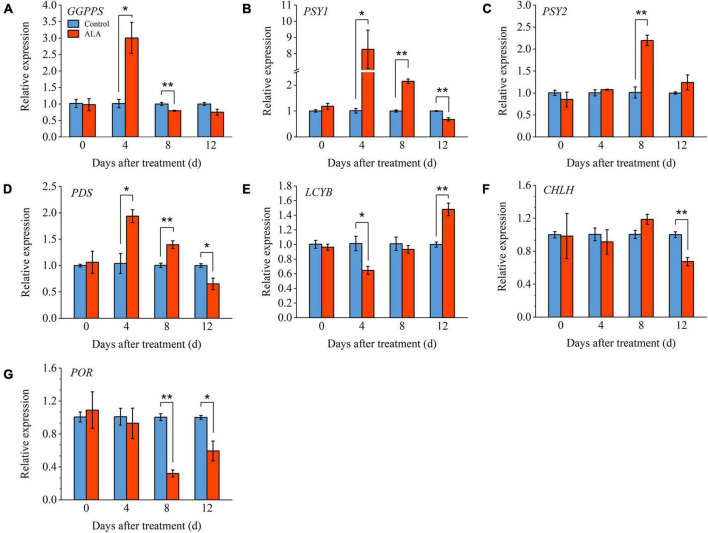
Effects of ALA on relative expression levels of key genes involved in the carotenoids metabolic and chlorophyll biosynthesis pathways in postharvest tomato fruit. **(A)** Relative gene expression of *geranylgeranyl diphosphate synthase* (*GGPPS*); **(B)** relative gene expression of *phytoene synthase 1* (*PSY1*); **(C)** relative gene expression of *phytoene synthase 2* (*PSY2*); **(D)** relative gene expression of *phytoene desaturase* (*PDS*); **(E)** relative gene expression of *lycopene*β*-cyclase* (*LCYB*); **(F)** relative gene expression of *Mg-chelatase* (*CHLH*); and **(G)** relative gene expression of *protochlorophyllide oxidoreductase* (*POR*). Vertical bars represent mean ± SE from three independent replicates, and asterisks indicate a significant difference between the treatments using the Student’s *t*-test (*p < 0.05, **p < 0.01). The control group was used as a reference at each sampling time point.

In order to further verify the mechanism of ALA-induced phenotypes, we measured the relative expression levels of key genes (*GGPPS*, *PSY1*, *PSY2*, *PDS*, and *LCYB*) involved in carotenoids metabolic pathway (Figure 6). Compared with control, the expression level of *GGPPS* was significantly upregulated in the ALA-treated fruit and reached its highest level of 4 DAT (Figure 6A). The *PSY1* expression level in ALA-treated fruit was significantly upregulated from 4 to 8 DAT (Figure 6B). However, at 12 DAT, the expression level of *PSY1* was significantly downregulated in the ALA-treated fruit as compared to the control. The expression level of *PSY2* in ALA-treated fruit upregulated 8 DAT (Figure 6C). The expression pattern of the above genes was consistent with the trend of lycopene content. Compared with the control, ALA treatment significantly upregulated the expression level of *PDS* in tomato fruit at 4 and 8 DAT (Figure 6D). Moreover, by 12 DAT, the expression levels of *LCYB* in fruit treated with ALA were significantly higher than control groups (Figure 6E).

Under ALA treatment, the level of *CHLH* was downregulated significantly compared to untreated tomato fruit at 12 DAT (Figure 6F). The expression level of *POR* was significantly inhibited by ALA treatment at the 8 and 12 DAT (Figure 6G). Compared with the control group, its expression levels were downregulated by 2.13-fold and 0.69-fold with ALA treatment.

### Correlation analysis and principal component analysis

**FIGURE 7:**
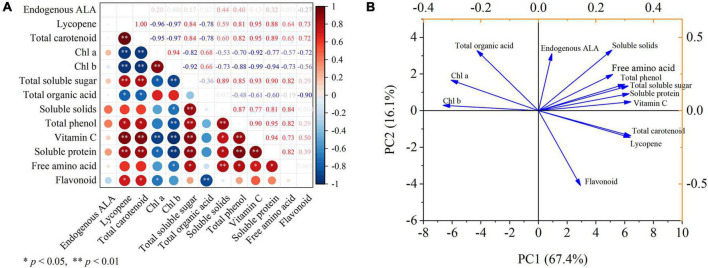
Correlation analysis and principal component analysis. **(A)** The heat map shows the correlation analysis between the observed parameters processed by ALA; **(B)** the score plot and loading plot show the PCA analysis of the observation parameters processed by ALA. Pearson correlation analysis and PCA was carried out with endogenous ALA, lycopene, total carotenoid, Chl *a*, Chl *b*, total soluble sugar, total organic acid, soluble solids, total phenol, vitamin C, soluble protein, free amino acid, and flavonoid as variables. * and ** denote correlation coefficients that are significant at *p* < 0.05 and 0.01 levels, respectively.

The correlation of the key parameters observed in this study was analyzed, and the results are displayed through the correlation heat map (Figure 7A). Lycopene had significantly correlated with total carotenoid, total soluble sugar, vitamin C, and soluble protein at *p* < 0.01 after ALA treatment, and negatively correlated with Chl *a*, Chl *b* (*p* < 0.01). Similarly, total carotenoid had significantly correlated with total soluble sugar, vitamin C, and soluble protein at *p* < 0.01 after ALA treatment, and had a negatively correlated with Chl *a*, Chl *b* (*p* < 0.01), and negatively correlated with total organic acid at *p* < 0.05. After ALA treatment, Chl *a* was significantly correlated with Chl *b* (*p* < 0.01), which was significantly negatively correlated with vitamin C (*p* < 0.01), total soluble sugar, and soluble protein (*p* < 0.05). Soluble solids had significantly correlated with total soluble sugar, total phenol, and free amino acid at *p* < 0.01 by ALA treatment. After ALA treatment, the total phenol had significantly correlated with total soluble sugar, soluble solids, vitamin C, and soluble protein at *p* < 0.01, and negatively correlated with Chl *b* (*p* < 0.01). Moreover, flavonoid had a significantly negative correlation with total organic acid (*p* < 0.01).

The sum of the first two principal components treated with ALA reached 83.5% (Figure 7B). PC1 accounted for 67.4% of the total variance and PC2 accounted for 16.1% of the total variance. In addition, it can be seen from the loading plot that total carotenoid and soluble solids showed strong loadings with the first and second principal components, thus can be used as representative factors to reflect the effects of ALA on the external color and internal nutritional quality of tomato fruit.

## Discussion

As one of the standards for judging maturity, the change in fruit firmness involves many physical properties, such as cell wall structure and cuticle properties (30, 31). The degradation of the primary cell wall during fruit ripening leads to texture changes and reduced firmness (32). Previous studies have found that rhizospheric ALA application significantly reduced the firmness of “Fuji” apple (21). In this study, ALA treatment accelerated the tomato fruit softening during postharvest storage. Compared with control, the firmness of ALA-treated fruit was markedly lower at 8 DAT. At 12 DAT, the fruit firmness became lower because the fruit treated with ALA was over-ripeness (Table 1). In recent years, some studies have shown that ALA can increase the contents of vitamin C, soluble solids, and soluble protein in horticultural crops such as apples and grapes (21, 33). In this study, similar effects were observed, at 8 DAT, the contents of soluble solids, vitamin C, free amino acid, and soluble protein in ALA-treated fruit were significantly higher than the control (Table 1). These results showed that ALA could improve the internal nutritional qualities of tomato fruit and accelerate fruit ripening during postharvest storage. Combining the color parameters with other quality indexes, we considered that fruit in control group matured at 12 DAT, whereas fruit under ALA treatment have become over-ripeness at 12 DAT.

The components of soluble sugars and organic acids and their proportion are crucial in the formation of tomato fruit flavor quality (34). The result of previous study showed that the application of ALA promoted the accumulation of photosynthetic products such as starch and sugar, thus improving the quality of crops (35). After irrigation with an appropriate concentration of ALA solution, the content of total soluble sugar in apple fruit was increased, and the content of titratable acid was reduced, which lead to the increase in sugar–acid ratio (21). In this study, under ALA treatment, the total soluble sugar content was increased, and the content of organic acid decreased, resulted in the increase of sugar-acid ratio in fruit at 8 DAT, these were consistent with the above research results (Figures 1J-L). It is important to note that the sweetness of most fruit is determined by three main sugars, namely, fructose, glucose, and sucrose, which are critical factors for taste characteristics and organoleptic quality (36, 37). In this study, ALA treatment increased fructose, glucose, and sucrose content of tomato fruit at 8 DAT (Figures 1A-C). Previous studies have found that the application of ALA in *Agrostis stolonifera* enhanced the abundance of glucose and fructose and could improve energy reserves and carbon metabolism (38). This result indicated that ALA had a positive effect on the transformation and accumulation of sugar components in tomato fruit. Organic acid content in tomato fruit was highly dependent on maturity (39), fruit acidity is produced by the total non-volatile acids and stored in the vacuoles of cells in the form of free organic acids (40). Citric acid and malic acid are dominant organic acid components accumulated in mature tomato fruit (41), which determine the acidity of fruit (42). In this study, citric acid content was the highest during tomato ripening, and it always dominated by the organic acid components. The contents of quinic acid (Figure 1E), malic acid (Figure 1F), citric acid (Figure 1H), and total organic acid (Figure 1K) in fruit with ALA treatment was significantly lower than that in the control at 8 DAT. These results showed that the acidity of tomato fruit was reduced after ALA treatment. It has been reported that the concentrations of organic acids fell with tomato fruit over-ripening (43). The tomato fruit treated with ALA was over-ripening at 12 DAT, the content of tartaric acid (Figure 1D), quinic acid (Figure 1E), malic acid (Figure 1F), shikimic acid (Figure 1G), citric acid (Figure 1H), and succinic acid (Figure 1I) of tomato fruit was decreased significantly, and the content of total organic acid (Figure 1K) was significantly decreased. Agius et al. (44) also reported similar results, during tomato fruit ripening, citric acid is the dominant organic acid, at mature green stage, and there is a lot of malic acid in the fruit while its content in ripe fruit is fairly low. At 8 and 12 DAT, ALA treatment increased total soluble sugar content, decreased total organic acid content, and increased sugar-acid ratio. These results further indicated that ALA could improve the tomato fruit flavor quality.

Secondary metabolites phenolic acids and flavonoids play essential roles in human health by scavenging the free radicals from cell metabolism to avoid the oxidative damage that leads to aging and age-related diseases (5, 45) and are important substances that regulate the quality of fruit and vegetables (46). Previous studies indicated that the application of ALA significantly increased the accumulation of total phenol in *Ginkgo biloba* leaves (47). Here, we found that ALA treatment markedly increased total phenol and flavonoid content in tomato fruit at 8 and 12 DAT (Figure 2). In addition, our study results indicated that the contents of five phenolic acids and two flavonoid compounds in ALA-treated fruit were significantly increased, including protocatechuic acid, gentianic acid, p-hydroxybenzoic acid, caffeic acid, cynarin, quercetin, and rutin (Figure 3). This further indicates that the positive effect of ALA on tomato fruit quality is closely related to the regulation of the accumulation of bioactive substances contents, such as phenolic acid and flavonoid compounds (48, 49).

As a natural plant growth regulator, the role of ALA in regulating ripening and promoting color of tomato fruit has been confirmed recently (22). It has been found that ALA markedly advanced the harvest time of grapes (33). The anthocyanin content of fruit skin was significantly increased by the appropriate concentration of ALA treated on apple fruit, thereby improving color of fruit (50). In the present study, the ALA treatment induced significant color changes in tomato fruit, and at 4 and 8 DAT, the a* value of the ALA-treated fruit was significantly higher than that of the control (Figure 4).

The coloring of red tomato is mainly due to the accumulation of lycopene (51). The previous study has shown that ALA could regulate the accumulation of lycopene (52). In the present study, the content of phytoene in ALA-treated fruit was markedly higher than that of the control at 4 and 8 DAT but decreased at 12 DAT. At 8 DAT, the content of lycopene in tomato fruit treated with ALA was markedly higher than that of the control. Moreover, the content of total carotenoids in tomato fruit increased significantly after ALA treatment (Figure 5F). These results indicated that ALA could promote the conversion of phytoene to lycopene and increase the synthesis of lycopene and other carotenoids downstream, thus promoting the transformation of fruit color. In addition, the expression level of *GGPPS* was upregulated at 4 DAT, while downregulated at 8 DAT (Figure 6A). GGPPS, the first enzyme to catalyze the reaction of precursor substances during lycopene synthesis, is abundantly expressed during fruit ripening (53). The study has shown that the step from GGPP to phytoene is crucial to increase the level of carotenoids, that is, PSY is a key rate-limiting enzyme in the synthesis and metabolism of carotenoids (54). In the mutant which suppressed *PSY1*, the synthesis and accumulation of lycopene and other carotenoids were prevented, and the tomato fruit displays a characteristic yellow phenotype (55). *PDS* is one of the driving forces to promote the conversion from phytoene to lycopene (56). In this study, the expression levels of *PSY1* and *PDS* in fruit with ALA treatment were significantly upregulated from 4 to 8 DAT. The *PSY2* expression level in fruit with ALA treatment was upregulated at 8 DAT (Figures 6B-D). These results have shown that ALA could promote lycopene accumulation by stimulating the expression of the lycopene biosynthetic key genes, including *PSY1*, *PSY2*, and *PDS*. Therefore, the positive role of ALA on carotenoid synthesis and accumulation in tomato fruit is probably caused by the expression of key genes in carotenoid synthesis induced by ALA. Besides, the degradation of lycopene is catalyzed by β-cyclase (LCYB) and ε-cyclase (LCY-E), then produce β-carotene and α-carotene, respectively (57). Studies have shown that the biosynthesis of lutein and β-carotene in tomato and citrus is regulated by *LCYB* (58, 59). In this study, ALA significantly increased the expression level of *LCYB* at 12 DAT (Figure 6E); meanwhile, the β-carotene accumulated significantly (Figure 5E). This suggested that the fruit is over-ripeness at 12 DAT, and the lycopene starts to degrade intensively. Ethylene was the key factor leading to the ripening of climacteric fruit (60), and the rapid accumulation of carotenoids such as lycopene in tomato fruit is closely related to the increase in ethylene (61). Interestingly, it has been suggested that ALA can be used as a synthetic precursor of ethylene. Specifically, tomato pericarp was incubated in (2, 3-^3^H) ALA, and then, radioactivity from ^3^H-ALA could be detected in ethylene from the pericarp tissue (62). Other studies have shown that the application of ALA could increase the synthesis of ethylene in plant tissues. For example, ethylene production was significantly increased in pink-stage tomato fruit skin by soaking in ALA solution compared with the control (63). ALA treatment upregulated the expression levels of key genes of ethylene biosynthesis (*ACS1* and *ACO4*), and ethylene signal transduction genes (*ETR2*, *EIN3-1*, and *EIN3-2*) in apple leaves, thus promoting the ethylene release (64). Therefore, we speculate that ALA promotes fruit ripening and lycopene accumulation by activating ethylene synthesis. However, the specific mechanism has not been elucidated yet and requires further investigation.

The biosynthesis and metabolism of ALA is a momentous bioprocess in higher plants, and it is called Beale pathway or C_5_ pathway (Figure 5A). Glutamate from the tricarboxylic acid (TCA) cycle is a precursor of ALA synthesis; it ligates tRNA*^Glu^* and generates L-glutamy-tRNA. Then, ALA is finally generated through reduction and transamination reactions (14, 65, 66). Starting from Proto-IX, the metabolism of ALA is divided into two branches, one is the Fe-branch which produces heme, and the other is the Mg-branch, which finally produce chlorophyll under the catalysis of Mg-chelatase (CHLH) and protochlorophyllide oxidoreductase (POR) (67). It is worth mentioning that feedback inhibition plays an important regulative role in ALA metabolic pathway (68, 69). ALA can not only be used as a precursor of chlorophyll synthesis (13) but also its metabolism is closely related to fruit ripening (22). The expression level of *GSAT* was downregulated gradually during tomato fruit ripening, as well as the *ALAD* (5-aminolevulinic acid dehydratase, the first enzyme in downstream of ALA metabolism pathway); meanwhile, the content of chlorophyll decreased significantly when the content of lycopene increased (70, 71). In this study, the content of derivatives of ALA metabolic pathway increased at early storage time after being treated by exogenous ALA, however, decreased at the end of storage. The content of endogenous ALA was decreased in ALA-treated fruit with the passage of storage but significantly increased at the end of storage. Compared with the control, the contents of Chl *a* and Chl *b* were markedly decreased at 8 DAT (Figures 5I,J). Moreover, the expression levels of *CHLH* and *POR* were significantly inhibited by ALA treatment at the end of storage (Figures 6F,G). These results showed that the application of ALA could promote chlorophyll synthesis of tomato fruit in the early storage, while obstructing chlorophyll synthesis at the end of storage. Besides, the content of endogenous ALA increased at the end of storage, further indicating that the metabolism of ALA was inhibited after fruit coloring. Lycopene cyclase is the main driving force for catalyzing lycopene degradation, but after inhibiting the activity of lycopene cyclase, it was found that the metabolism of chlorophyll synthesis precursors (including Mg-PPIX, Mg-PPIX-Me, and PPIX) was obstructed (72). These results suggested that there might be some interaction regulative mechanism between the metabolism of ALA and biosynthesis of lycopene in tomato fruit, which should be deeply explored in further studies.

Principal component analysis is a statistical analysis method that converts multiple indicators into several comprehensive indicators and has become one of the main methods for the comprehensive quality evaluation of fruits and vegetables (73). The PCA method avoids bias caused by subjective factors, and the selected principal components should cover more than 80% of the total information in the original data (74). In the present study, total carotenoid and soluble solids showed strong loadings with the first and second principal components, thus can be used as representative factors to reflect the effects of ALA on the external color and internal nutritional and flavor quality of tomato fruit.

## Conclusion

**FIGURE 8:**
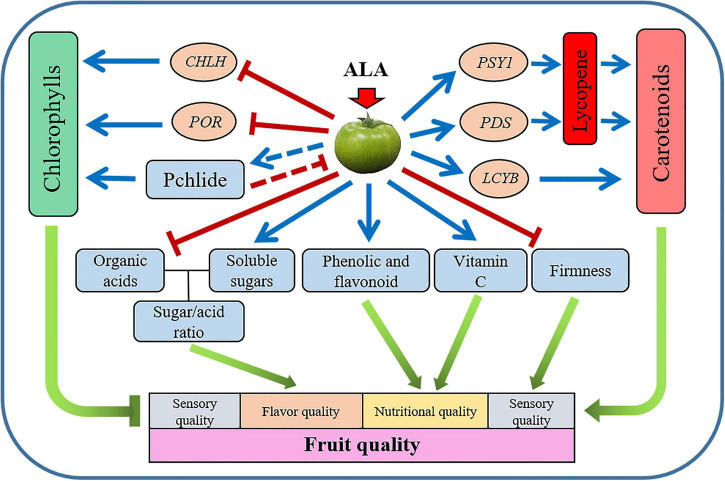
Schematic representation of the proposed model for the effect of ALA treatment on tomato fruit quality. Application of ALA negatively regulates chlorophyll biosynthesis by downregulating the expression of *CHLH* and *POR* genes. Application of ALA modulates carotenoid biosynthesis by the direct promotion of the expression of *PSY1*, *PDS*, and *LCYB* genes. ALA improved the quality of postharvest tomato fruit by regulating the contents of primary and secondary metabolites. The blue line segment with an arrow indicates promotion, the red line segment with a short line indicates inhibition, and the red dotted line with a short line indicates feedback inhibition.

The present study has demonstrated that during postharvest storage, the application of ALA on green mature tomato fruit significantly promoted coloring by stimulating carotenoid accumulation and suppressing chlorophyll synthesis. The mechanism might be the upregulation of the gene expressions of *GGPPS*, *PSY1*, *PDS*, and *LCYB*, as well as the downregulation of the gene expressions of *CHLH* and *POR*. Besides, the application of ALA increased the contents of vitamin C, free amino acids, soluble solids, and soluble protein in tomato fruit and decreased fruit firmness. In addition, the exogenous ALA also regulated the contents of primary and secondary metabolites during fruit ripening of postharvest tomatoes, such as sugar, organic acid, phenolic acid, and flavonoid compounds (Figure 8). The color development, flavor, and nutrient qualities of postharvest tomato fruit were earlier in the ALA-treated fruit than in the control group.

In summary, the exogenous application of ALA could not only promote fruit ripening but also improve the internal nutritional and flavor quality of postharvest tomato fruit.

## Data availability statement

The original contributions presented in this study are included in the article/Supplementary material, further inquiries can be directed to the corresponding authors.

## Author contributions

YW and JY designed and coordinated this study and revised the manuscript. JW and YW conducted data analysis and drafted the manuscript. JW and HY treated the samples. JZ helped with data analysis. BA, ZT, JX, JL, and WL reviewed and revised the manuscript. All authors contributed to the article and approved the submitted version.
