# Supplementary material for: Application of 5-aminolevulinic acid promotes ripening and accumulation of primary and secondary metabolites in postharvest tomato fruit
Source: Front Nutr. 2022 Nov 10;9:1036843. doi: 10.3389/fnut.2022.1036843 (PMC9686309; doi:10.3389/fnut.2022.1036843)
Supplement: Supplementary file 1 [file Table_1.DOCX]

**TABLE S1** Gradient elution conditions

| Time  (min) | Current Speed  (mL·min^-1^) | Mobile phase | |
| --- | --- | --- | --- |
|  |  | A  Methanol | B  1% Acetic acid |
| — | 1.1 | 5 % | 95 % |
| 30 | 1.1 | 30 % | 70 % |
| 40 | 1.1 | 40 % | 60 % |
| 45 | 1.1 | 50 % | 50 % |
| 50 | 1.1 | 70 % | 30 % |
| 55 | 1.1 | 40 % | 60 % |
| 57 | 1.1 | 5 % | 95 % |
| 60 | 1.1 | 5 % | 95 % |

**TABLE S2** The detection wavelength, retention time, and standards manufacturer of the phenolic acid and flavonoid compounds

| Compounds | Detection wavelength (nm) | Retention time (min) | Standards manufacturer |
| --- | --- | --- | --- |
| Protocatechuic acid | 240 | 11.3 | Acmec Biochemical Co., Ltd., China |
| P-hydroxybenzoic acid | 240 | 17.7 | Yuanye Bio-Technology Co., Ltd., China |
| Chlorogenic acid | 240 | 19.2 | Yuanye Bio-Technology Co., Ltd., China |
| rutin | 240 | 42.5 | Yuanye Bio-Technology Co., Ltd., China |
| Quercetin | 240 | 50.1 | Yuanye Bio-Technology Co., Ltd., China |
| Gallic acid | 280 | 5.9 | Yuanye Bio-Technology Co., Ltd., China |
| 4-Coumaric acid | 280 | 31.3 | Yuanye Bio-Technology Co., Ltd., China |
| Ferulic acid | 280 | 34.2 | Yuanye Bio-Technology Co., Ltd., China |
| Cinnamic acid | 280 | 49.5 | Yuanye Bio-Technology Co., Ltd., China |
| Naringin | 280 | 50.4 | Yuanye Bio-Technology Co., Ltd., China |
| Gentianic acid | 322 | 20.9 | Acmec Biochemical Co., Ltd., China |
| Caffeic acid | 322 | 23.2 | Yuanye Bio-Technology Co., Ltd., China |
| Cynarin | 322 | 27.3 | Yuanye Bio-Technology Co., Ltd., China |
| Benzoic acid | 322 | 38.6 | Yuanye Bio-Technology Co., Ltd., China |
| Kaempferol | 322 | 52.2 | Yuanye Bio-Technology Co., Ltd., China |

**TABLE S3** Primer sequences and Genebank accession number of *GGPPS, PSY1, PSY2, PDS*, *LCYB*, *CHLH*, *POR* and *Actin* gene sequences

| Gene Symbol | Accession number | Forward primer 5'-3' | Reverse primer 5'-3' |
| --- | --- | --- | --- |
| *GGPPS* | NM_001366706.1 | CTGCCTGTGCCTTAGAGATGGTTC | CCTCGTCGAGTTGTGTCATCATCC |
| *PSY1* | NM_001247883.2 | GCTGGAAGGGTGACCGATAAATGG | GTCACGCCTTTCTCTGCCTCATC |
| *PSY2* | NM_001247742.2 | AAGAAGCAAATCCAGAGGGCAAGG | ACCGGCCATCTACTAGCAGAACTC |
| *PDS* | NM_001247166.2 | CGAGGTCGTCTTCTTTGGGAACTG | CAATCTTCTGGTCGTGGCATGGG |
| *LCYB* | NM_001247297.2 | GAGTCGTTGGAATCGGTGGTACAG | CAACAGGAGCCGCAGCTAGTG |
| *CHLH* | XM_004236562.4 | GTGCTGGCATGATGGAGAAGAGG | GAGGTTCTGAACGAGGTTGGTTGG |
| *POR* | NM_001317974.1 | TGGACCTCGCCTCTCTTGACAG | CAGCAGCATTAGCAACCAACACG |
| *Actin* | NM_001330119.1 | TTGTGTTGGACTCTGGTGATGGTG | GACGGAGAATGGCATGTGGAAGG |
